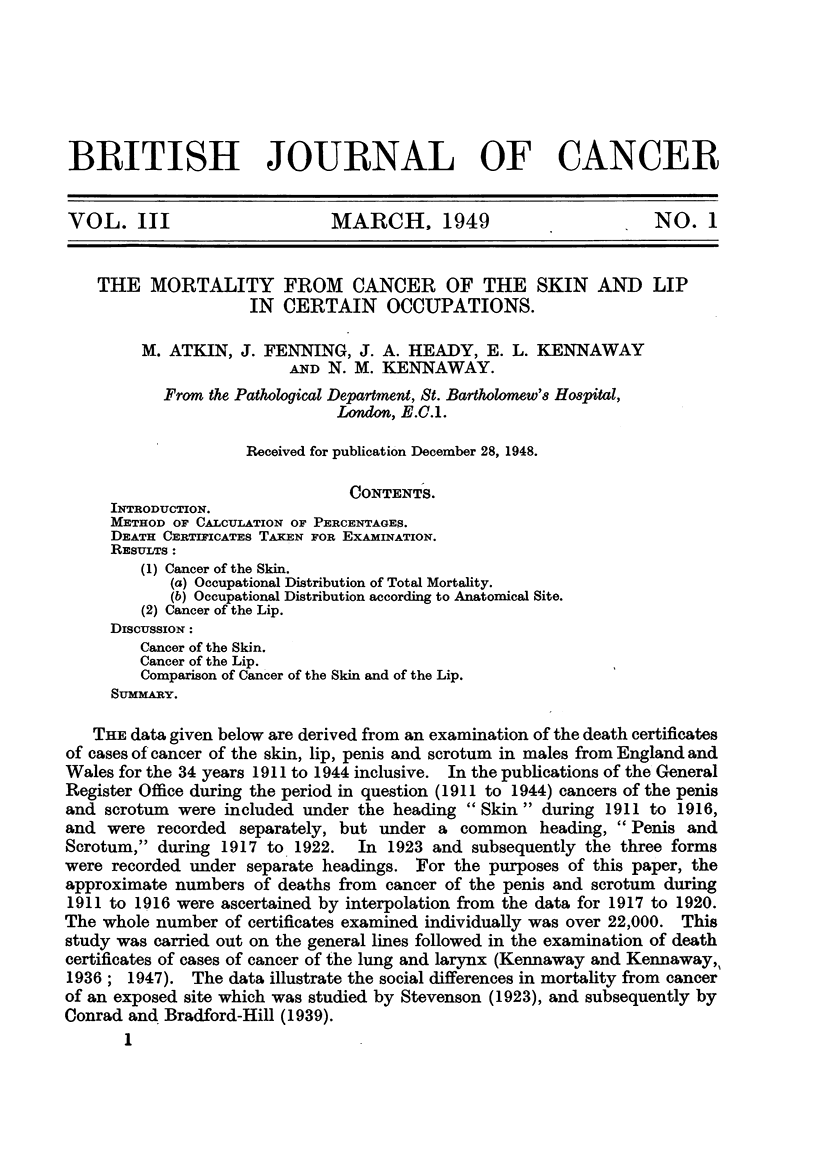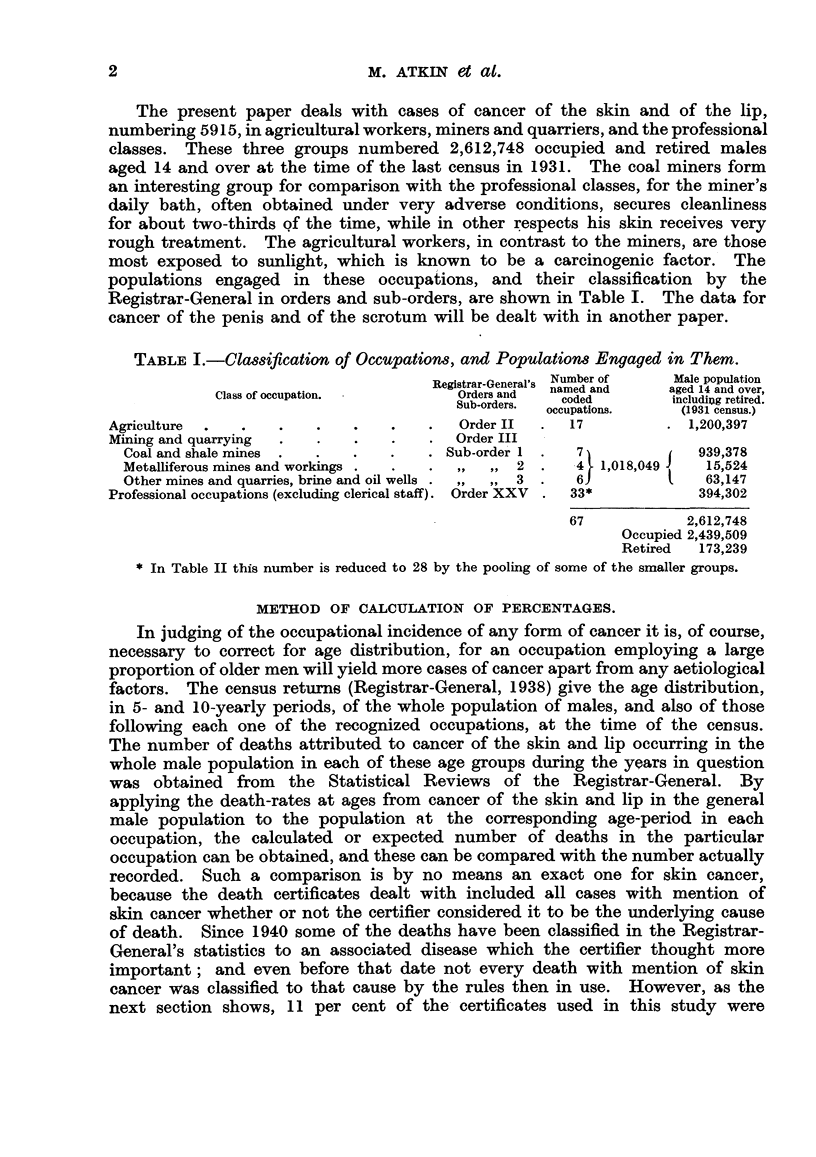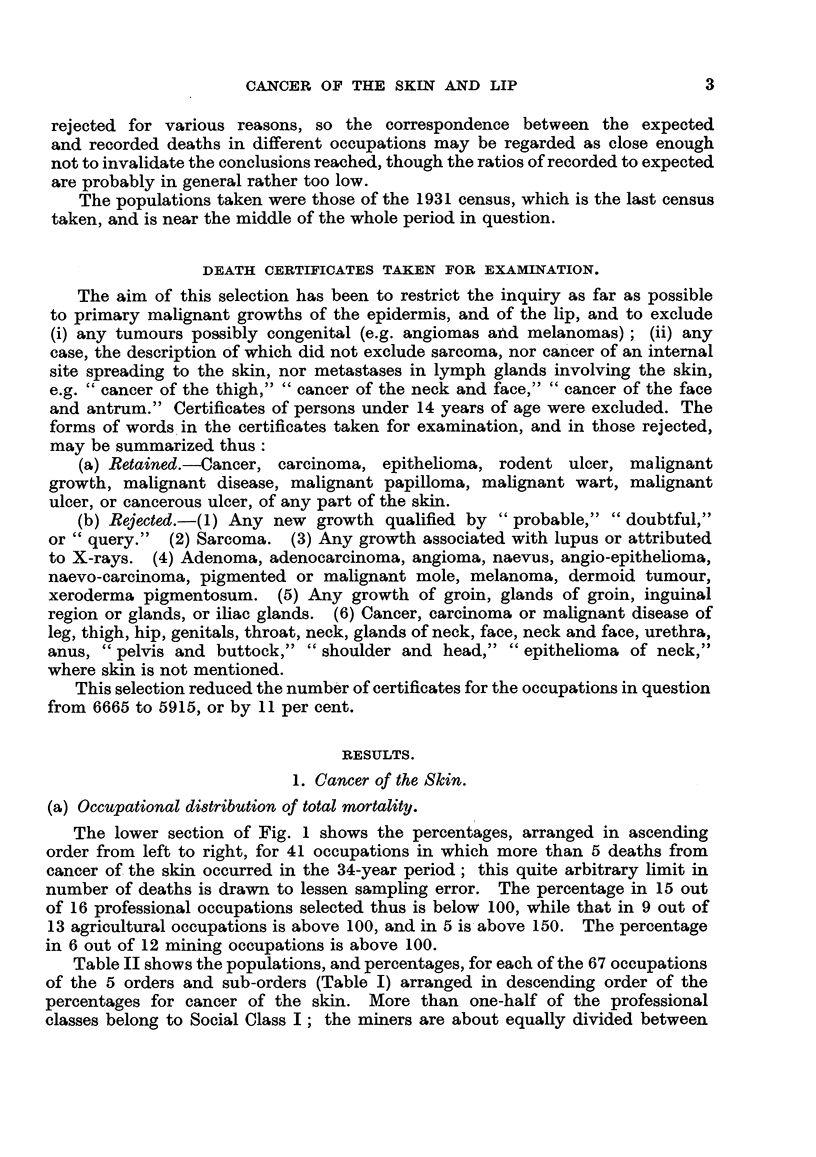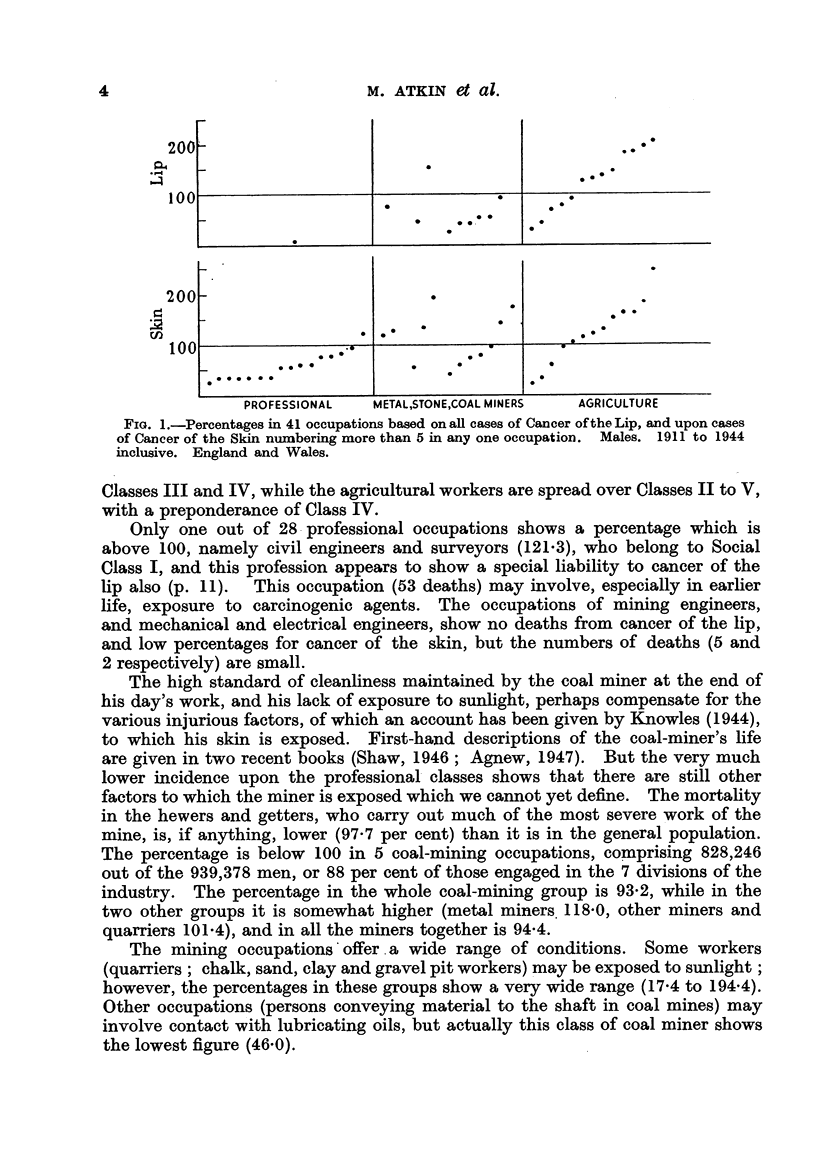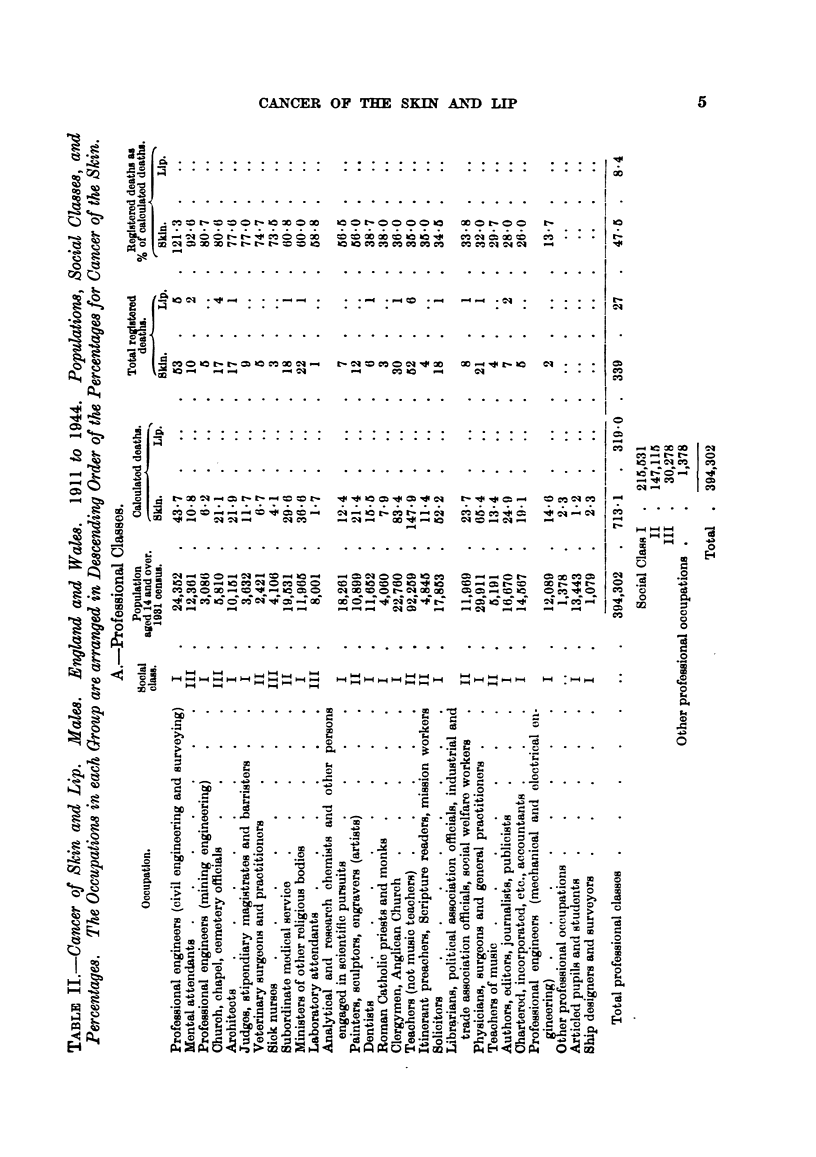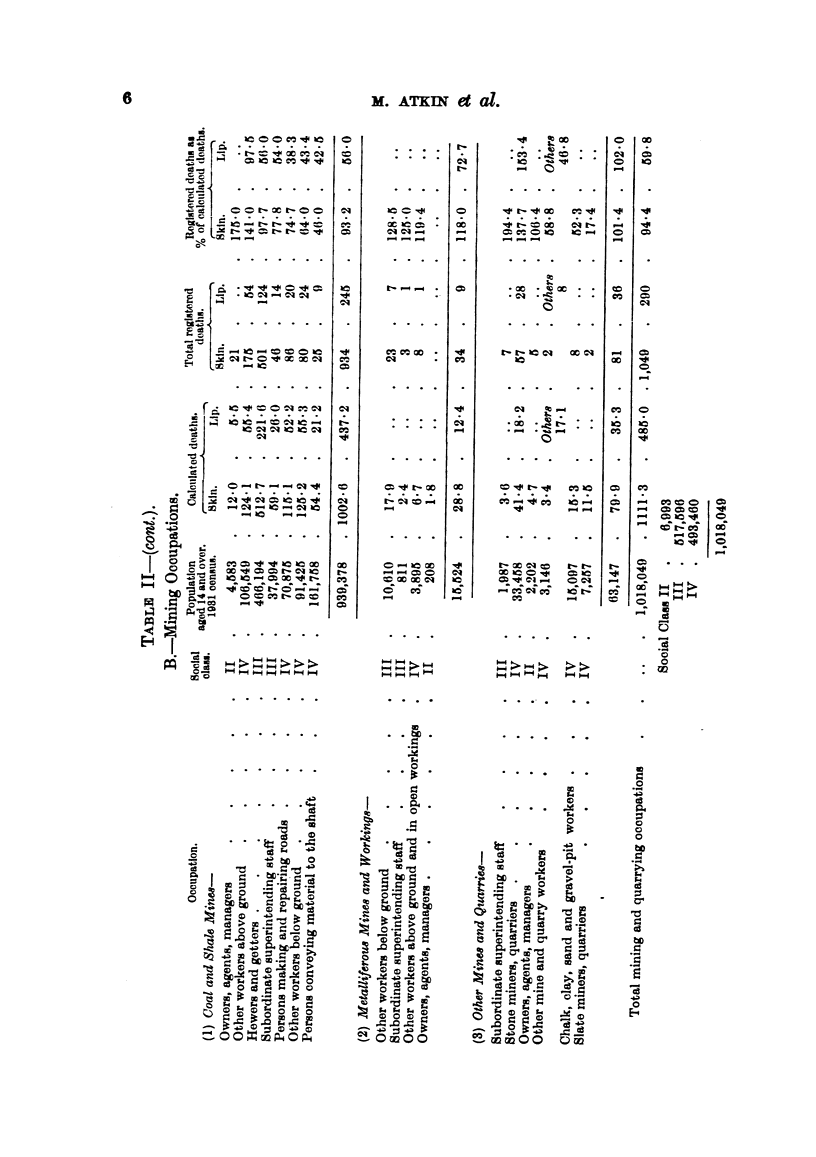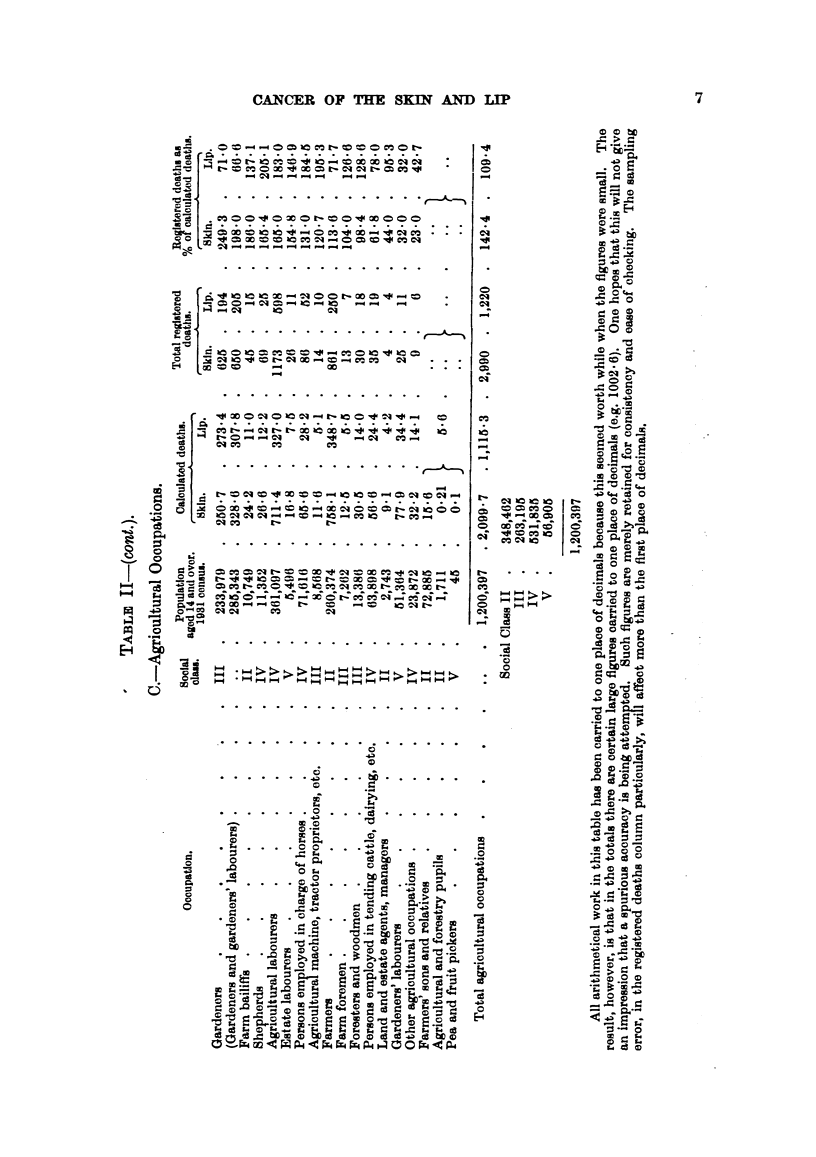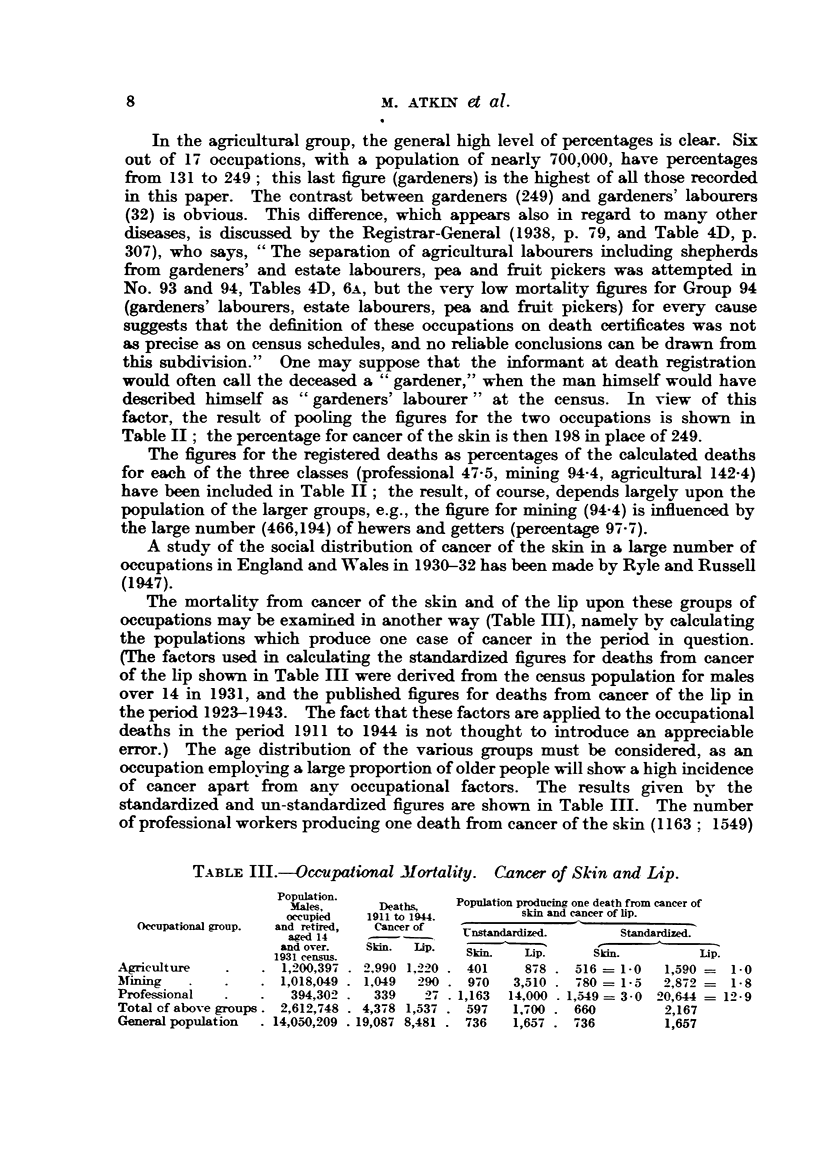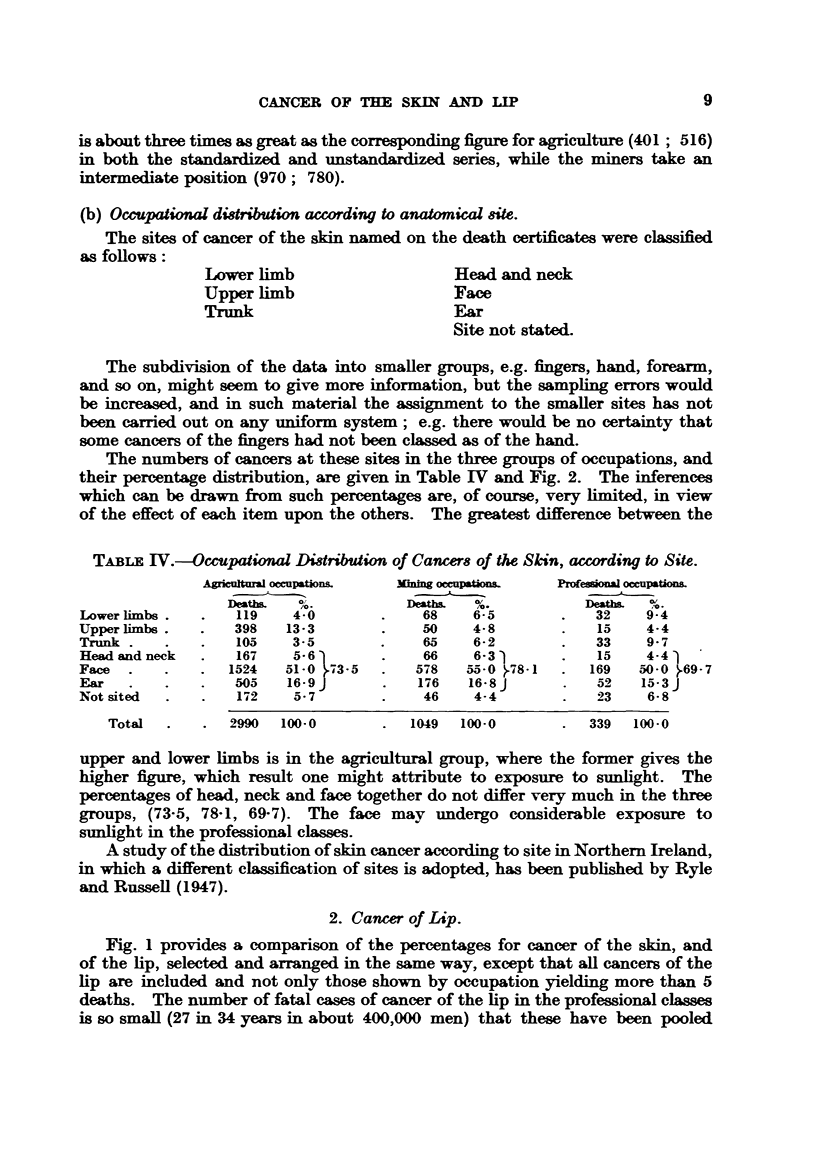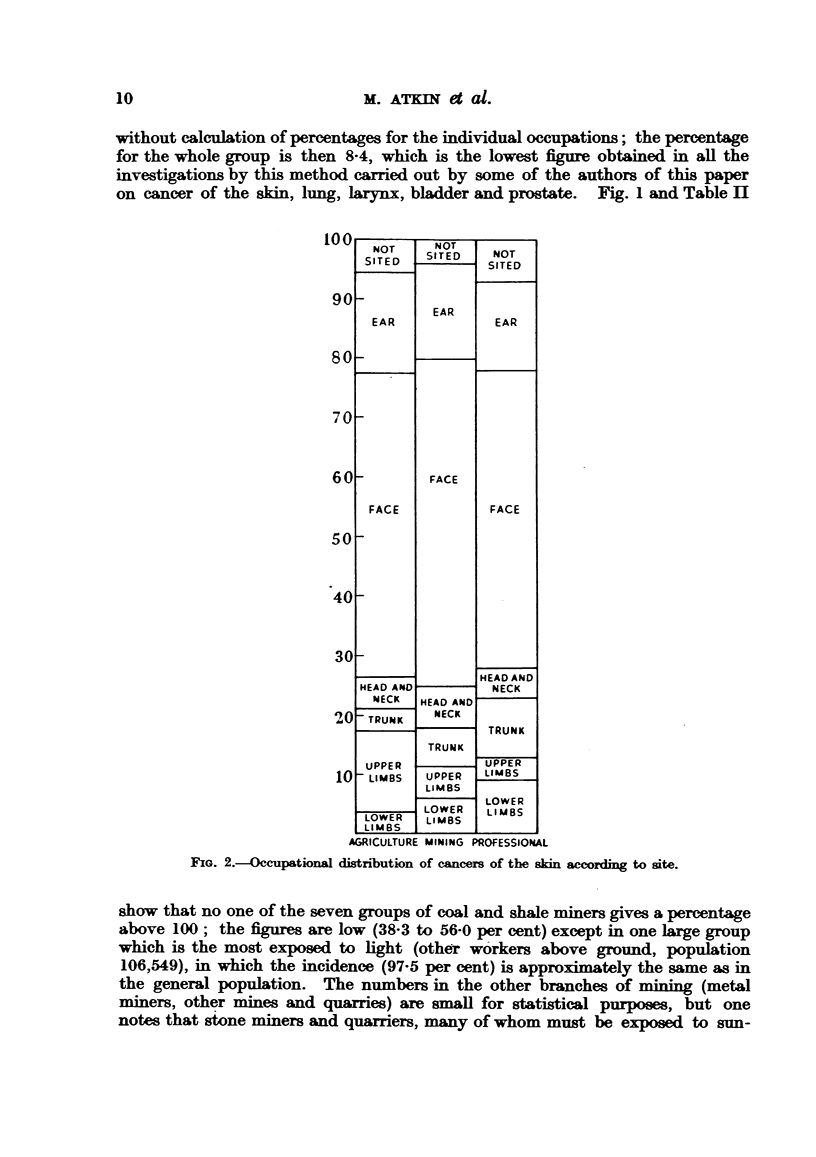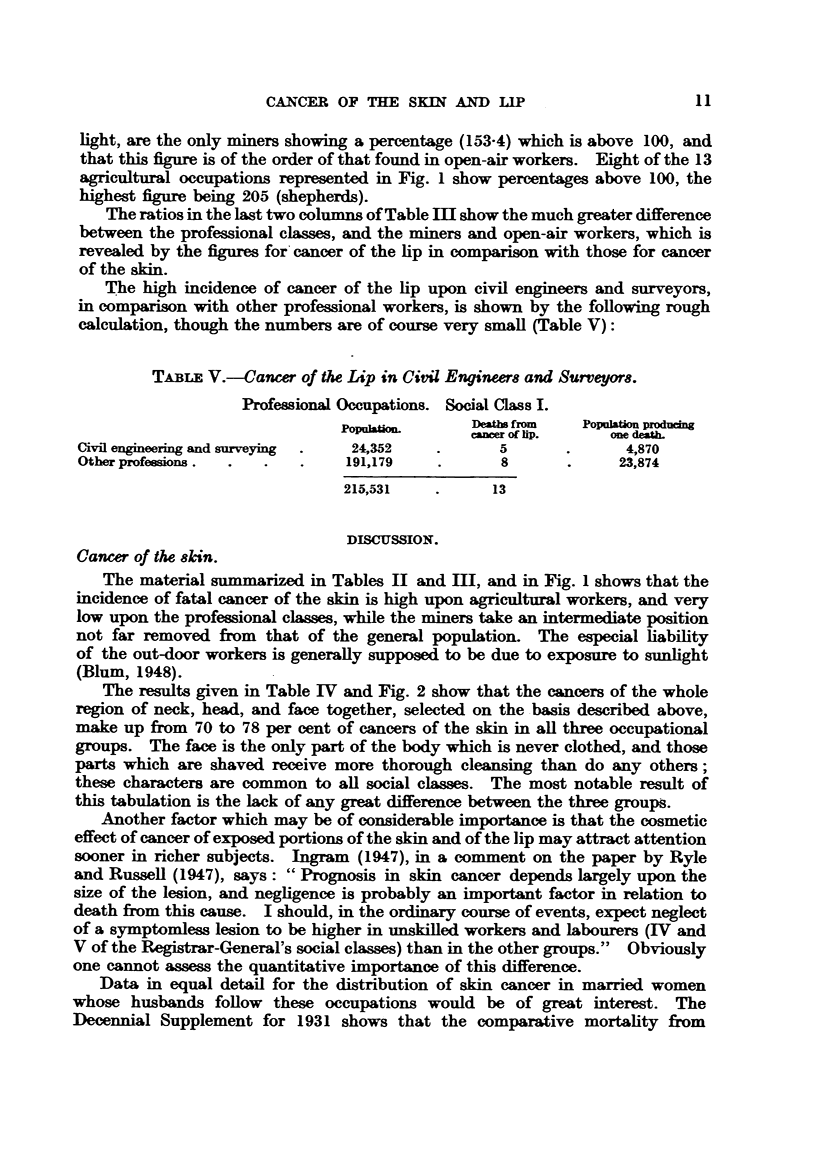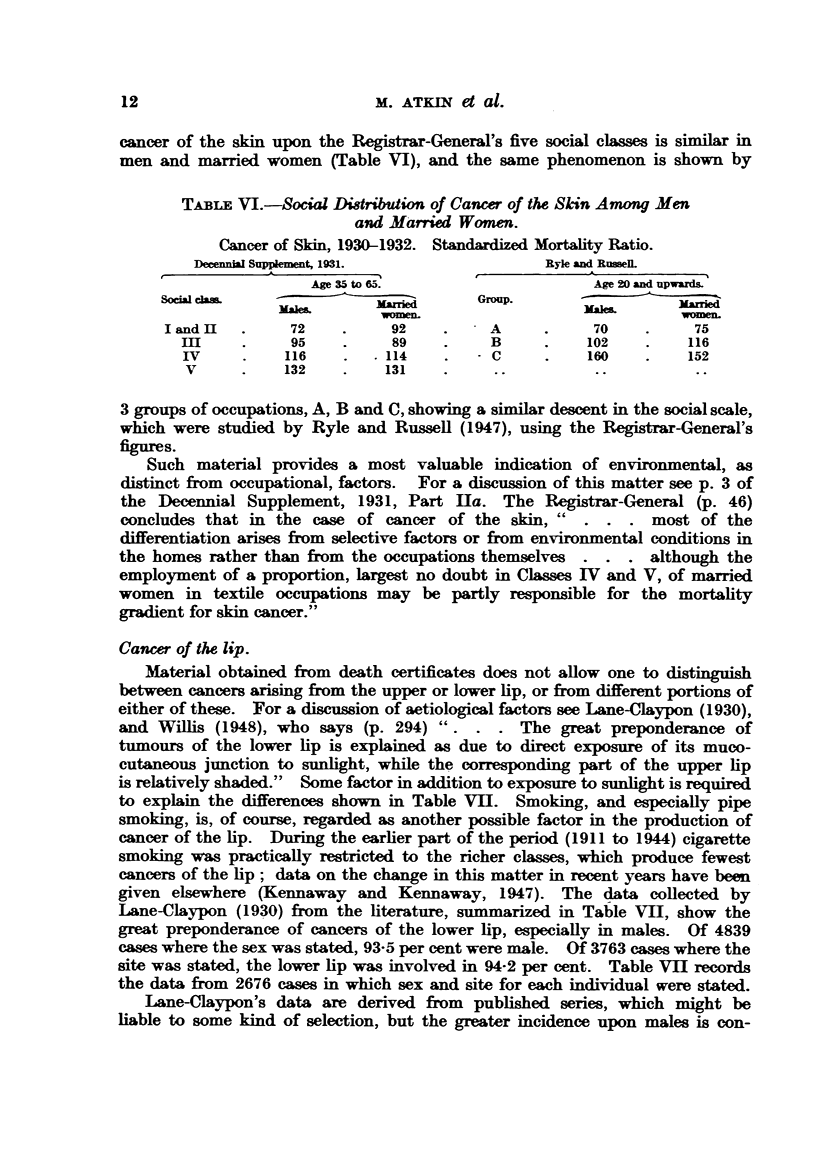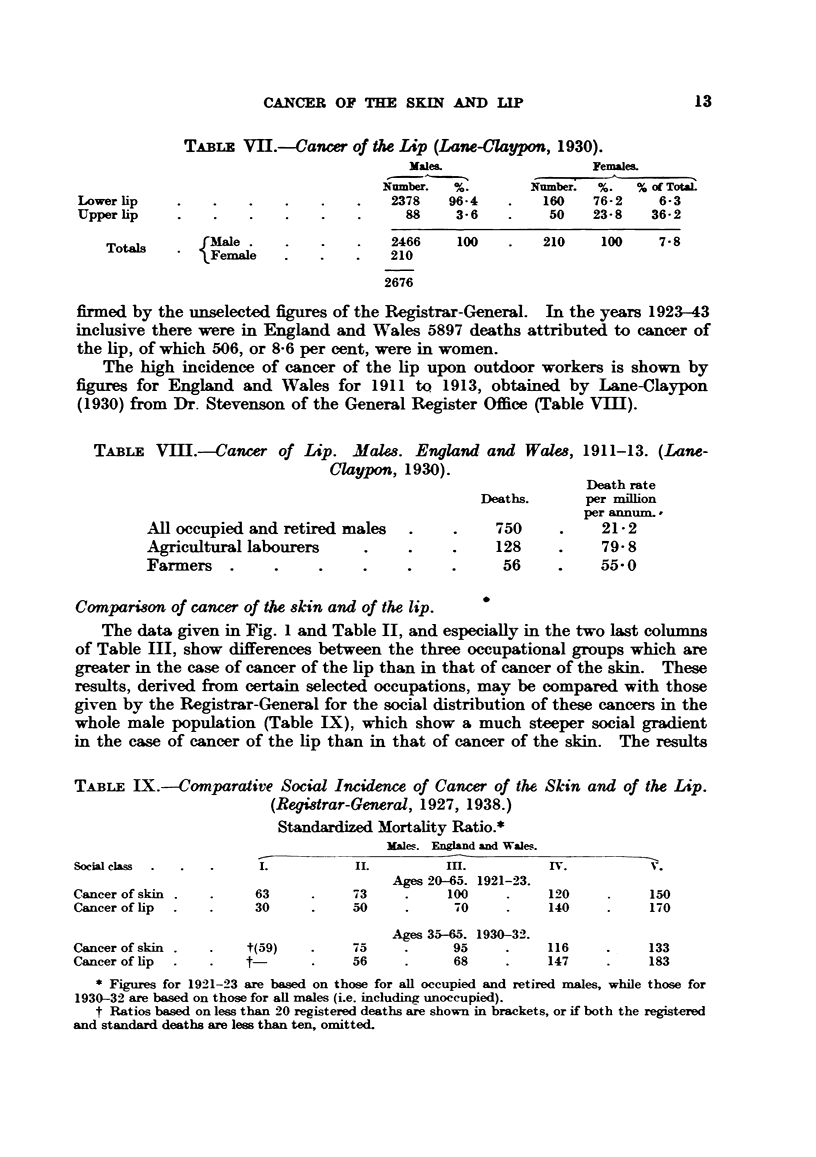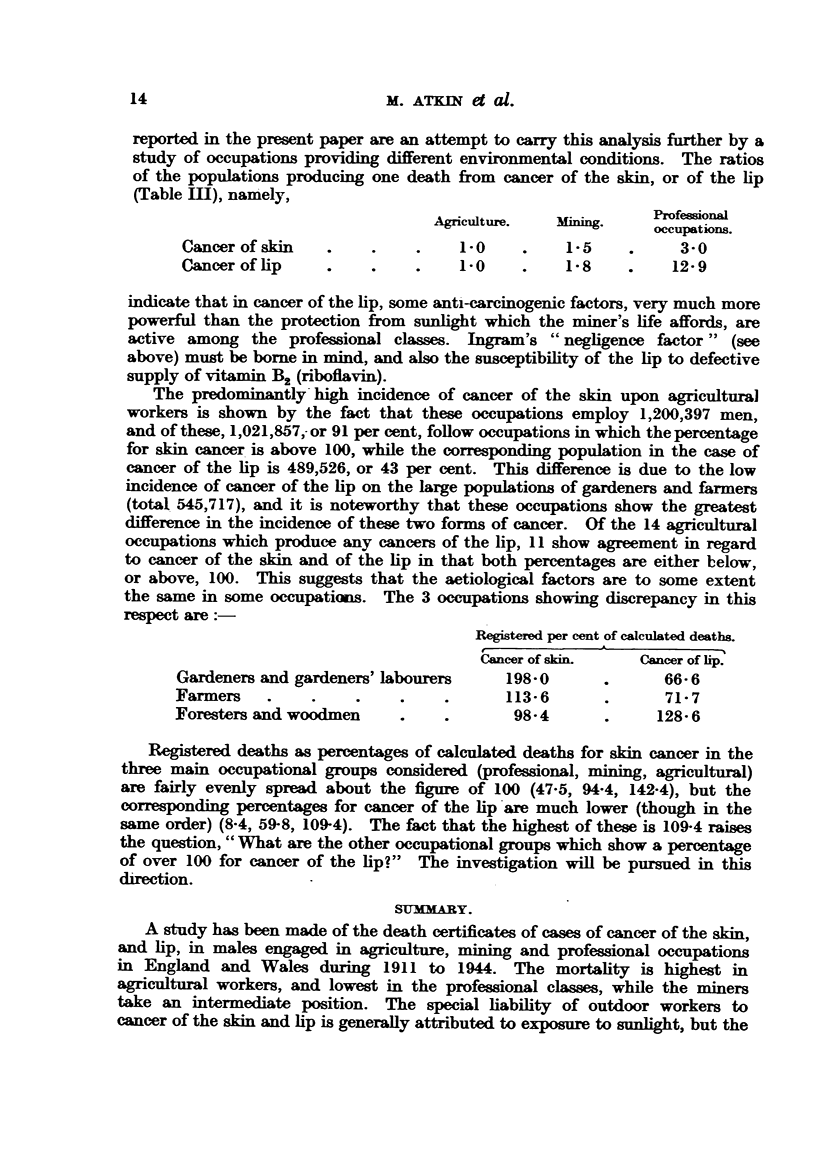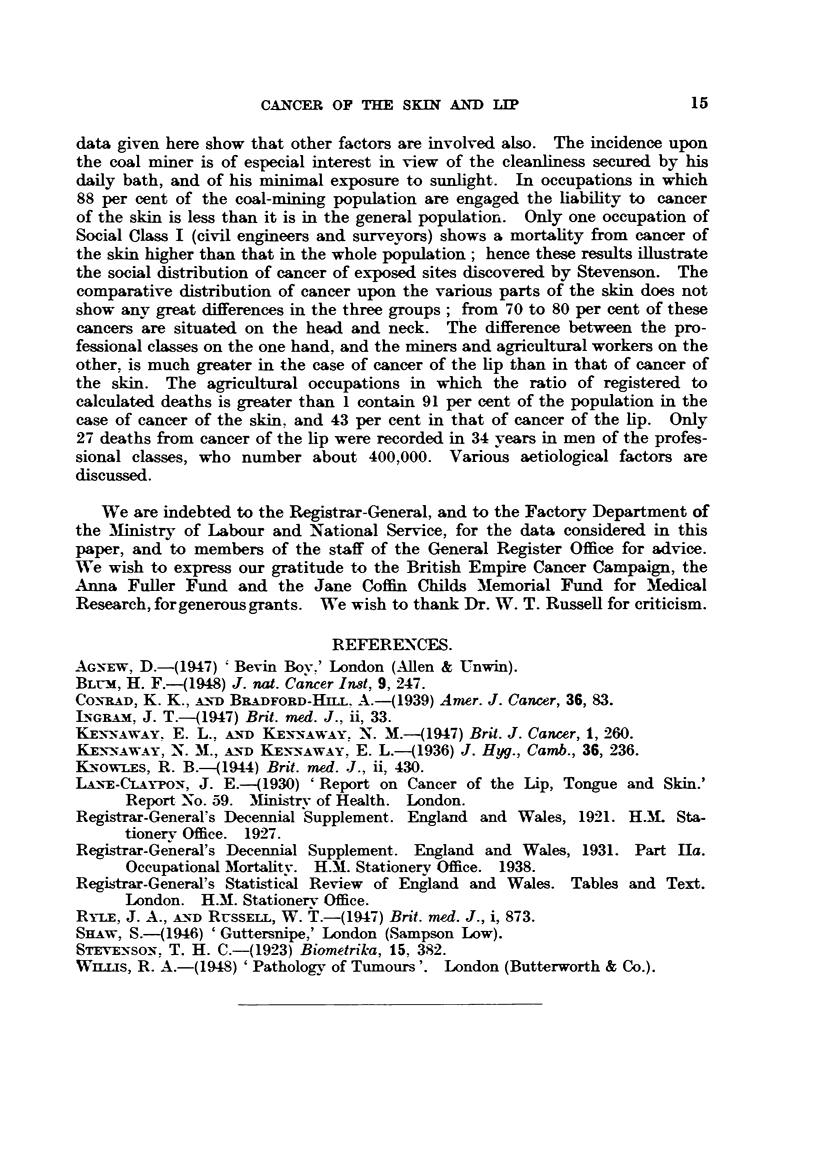# The Mortality from Cancer of the Skin and Lip in Certain Occupations

**DOI:** 10.1038/bjc.1949.1

**Published:** 1949-03

**Authors:** M. Atkin, J. Fenning, J. A. Heady, E. L. Kennaway, N. M. Kennaway


					
BRITISH JOURNAL OF CANCER

VOL. III              MARCH, 1949                 NO. 1

THE MORTALITY FROM CANCER OF THE SKIN AND LIP

IN CERTAIN OCCUPATIONS.

M. ATKIN, J. FENNING, J. A. HEADY, E. L. KENNAWAY

AND N. M. KENNAWAY.

From the Pathological Department, St. Bartholomew's Hospital,

London, E.G.1.

Received for publication December 28, 1948.

CONTENTS.
INTRODUCTION.

METHOD OF CALCULATION OF PERCENTAGES.

DEATH CERTIFICATES TAKEN FOR EXAMINATION.
RESULTS:

(1) Cancer of the Skin.

(a) Occupational Distribution of Total Mortality.

(b) Occupational Distribution according to Anatomical Site.
(2) Cancer of the Lip.

DISCUSSION:

Cancer of the Skin.
Cancer of the Lip.

Comparison of Cancer of the Skin and of the Lip.
SUMMARY.

THE data given below are derived from an examination of the death certificates
of cases of cancer of the skin, lip, penis and scrotum in males from England and
Wales for the 34 years 1911 to 1944 inclusive. In the publications of the General
Register Office during the period in question (1911 to 1944) cancers of the penis
and scrotum were included under the heading "Skin" during 1911 to 1916,
and were recorded separately, but under a common heading, "Penis and
Scrotum," during 1917 to 1922. In 1923 and subsequently the three forms
were recorded under separate headings. For the purposes of this paper, the
approximate numbers of deaths from cancer of the penis and scrotum during
1911 to 1916 were ascertained by interpolation from the data for 1917 to 1920.
The whole number of certificates examined individually was over 22,000. This
study was carried out on the general lines followed in the examination of death
certificates of cases of cancer of the lung and larynx (Kennaway and Kennaway,,
1936; 1947). The data illustrate the social differences in mortality from cancer
of an exposed site which was studied by Stevenson (1923), and subsequently by
Conrad and Bradford-Hill (1939).

1

M. ATKIN et at.

The present paper deals with cases of cancer of the skin and of the lip,
numbering 5915, in agricultural workers, miners and quarriers, and the professional
classes. These three groups numbered 2,612,748 occupied and retired males
aged 14 and over at the time of the last census in 1931. The coal miners form
an interesting group for comparison with the professional classes, for the miner's
daily bath, often obtained under very adverse conditions, secures cleanliness
for about two-thirds Qf the time, while in other respects his skin receives very
rough treatment. The agricultural workers, in contrast to the miners, are those
most exposed to sunlight, which is known to be a carcinogenic factor. The
populations engaged in these occupations, and their classification by the
Registrar-General in orders and sub-orders, are shown in Table I. The data for
cancer of the penis and of the scrotum will be dealt with in another paper.

TABLE I.-Classification of Occupations, and Populations Engaged in Them.

Registrar-General's Number of  Male population
Class of occupation.         SbOrders.    oe            19 andu

Cla.Orders and  named and  aged 14 and over,
Classof .Sub-orders.  coded  includi!g retired.

occupations.    (1931 census.)

Agriculture  .  .    .   .   .    .   .   Order II  .  17          . 1,200,397
Mining and quarrying  .  .   .    .   .   Order III

Coal and shale mines  .  .  .   .    . Sub-order 1 .  7              939,378
Metalliferous mines and workings .  .  .,,,,   2 .    4 1,018,049     15,524
Other mines and quarries, brine and oil wells .,,,,  3 .  6           63,147
Professional occupations (excluding clerical staff). Order XXV  .  33*  394,302

67            2,612,748

Occupied 2,439,509
Retired  173,239
* In Table II this number is reduced to 28 by the pooling of some of the smaller groups.

METHOD OF CALCULATION OF PERCENTAGES.

In judging of the occupational incidence of any form of cancer it is, of course,
necessary to correct for age distribution, for an occupation employing a large
proportion of older men will yield more cases of cancer apart from any aetiological
factors. The census returne (Registrar-General, 1938) give the age distribution,
in 5- and 10-yearly periods, of the whole population of males, and also of those
following each one of the recognized occupations, at the time of the census.
The number of deaths attributed to cancer of the skin and lip occurring in the
whole male population in each of these age groups during the years in question
was obtained from the Statistical Reviews of the Registrar-General. By
applying the death-rates at ages from cancer of the skin and lip in the general
male population to the population at the corresponding age-period in each
occupation, the calculated or expected number of deaths in the particular
occupation can be obtained, and these can be compared with the number actually
recorded. Such a comparison is by no means an exact one for skin cancer,
because the death certificates dealt with included all cases with mention of
skin cancer whether or not the certifier considered it to be the underlying cause
of death. Since 1940 some of the deaths have been classified in the Registrar-
General's statistics to an associated disease which the certifier thought more
important; and even before that date not every death with mention of skin
cancer was classified to that cause by the rules then in use. However, as the
next section shows, 11 per cent of the certificates used in this study were

2

CANCER OF THE SKIN AND LIP

rejected for various reasons, so the correspondence between the expected
and recorded deaths in different occupations may be regarded as close enough
not to invalidate the conclusions reached, though the ratios of recorded to expected
are probably in general rather too low.

The populations taken were those of the 1931 census, which is the last census
taken, and is near the middle of the whole period in question.

DEATH CERTIFICATES TAKEN FOR EXAMINATION.

The aim of this selection has been to restrict the inquiry as far as possible
to primary malignant growths of the epidermis, and of the lip, and to exclude
(i) any tumours possibly congenital (e.g. angiomas anid melanomas); (ii) any
case, the description of which did not exclude sarcoma, nor cancer of an internal
site spreading to the skin, nor metastases in lymph glands involving the skin,
e.g. " cancer of the thigh," " cancer of the neck and face," " cancer of the face
and antrum." Certificates of persons under 14 years of age were excluded. The
forms of words in the certificates taken for examination, and in those rejected,
may be summarized thus:

(a) Retained.-Cancer, carcinoma, epithelioma, rodent ulcer, malignant
growth, malignant disease, malignant papilloma, malignant wart, malignant
ulcer, or cancerous ulcer, of any part of the skin.

(b) Rejected.-(1) Any new growth qualified by "probable," "doubtful,"
or "query." (2) Sarcoma. (3) Any growth associated with lupus or attributed
to X-rays. (4) Adenoma, adenocarcinoma, angioma, naevus, angio-epithelioma,
naevo-carcinoma, pigmented or malignant mole, melanoma, dermoid tumour,
xeroderma pigmentosum. (5) Any growth of groin, glands of groin, inguinal
region or glands, or iliac glands. (6) Cancer, carcinoma or malignant disease of
leg, thigh, hip, genitals, throat, neck, glands of neck, face, neck and face, urethra,
anus, "pelvis and buttock," "shoulder and head," "epithelioma of neck,"
where skin is not mentioned.

This selection reduced the number of certificates for the occupations in question
from 6665 to 5915, or by 11 per cent.

RESULTS.

1. Cancer of the Skin.
(a) Occupational di8tribution of total mortality.

The lower section of Fig. 1 shows the percentages, arranged in ascending
order from left to right, for 41 occupations in which more than 5 deaths from
cancer of the skin occurred in the 34-year period; this quite arbitrary limit in
number of deaths is drawn to lessen sampling error. The percentage in 15 out
of 16 professional occupations selected thus is below 100, while that in 9 out of
13 agricultural occupations is above 100, and in 5 is above 150. The percentage
in 6 out of 12 mining occupations is above 100.

Table II shows the populations, and percentages, for each of the 67 occupations
of the 5 orders and sub-orders (Table I) arranged in descending order of the
percentages for cancer of the skin. More than one-half of the professional
classes belong to Social Class I; the miners are about equally divided between

3

M. ATKIN et al.

200

,-

._,4

100

CL)
dQ
;;

*      *

0          0

0                         4,

0      0 0 -* 0     0

0            0
0            1                       I

2~~~~~~~~~~~~~~~~~~~~~~~~~~~00

p200  _____________           0

I                     0

2oG       0O***   *           .0

10   .--      *         *-

?tee        0 ?    ?

PROFESSIONAL   METAL,STONE,COAL MINERS  AGRICULTURE

FIa. 1.-Percentages in 41 occupations based on all cases of Cancer ofthe Lip, and upon cases
of Cancer of the Skin numbering more than 5 in any one occupation. Males. 1911 to 1944
inclusive. England and Wales.

Classes III and IV, while the agricultural workers are spread over Classes II to V,
with a preponderance of Class IV.

Only one out of 28 professional occupations shows a percentage which is
above 100, namely civil engineers and surveyors (121.3), who belong to Social
Class I, and this profession appears to show a special liability to cancer of the
lip also (p. 11). This occupation (53 deaths) may involve, especially in earlier
life, exposure to carcinogenic agents. The occupations of mining engineers,
and mechanical and electrical engineers, show no deaths from cancer of the lip,
and low percentages for cancer of the skin, but the numbers of deaths (5 and
2 respectively) are small.

The high standard of cleanliness maintained by the coal miner at the end of
his day's work, and his lack of exposure to sunlight, perhaps compensate for the
various injurious factors, of which an account has been given by Knowles (1944),
to which his skin is exposed. First-hand descriptions of the coal-miner's life
are given in two recent books (Shaw, 1946; Agnew, 1947). But the very much
lower incidence upon the professional classes shows that there are still other
factors to which the miner is exposed which we cannot yet define. The mortality
in the hewers and getters, who carry out much of the most severe work of the
mine, is, if anything, lower (97-7 per cent) than it is in the general population.
The percentage is below 100 in 5 coal-mining occupations, comprising 828,246
out of the 939,378 men, or 88 per cent of those engaged in the 7 divisions of the
industry. The percentage in the whole coal-mining group is 93.2, while in the
two other groups it is somewhat higher (metal miners. 118.0, other miners and
quarriers 101-4), and in all the miners together is 94.4.

The mining occupations offer a wide range of conditions. Some workers
(quarriers; chalk, sand, clay and gravel pit workers) may be exposed to sunlight;
however, the percentages in these groups show a very wide range (17.4 to 194.4).
Other occupations (persons conveying material to the shaft in coal mines) may
involve contact with lubricating oils, but actually this class of coal miner shows
the lowest figure (46.0).

I

I

I

I

4

CANCER OF THE SKIN AND LIP

]r.

..    . . .  .  .  .  . . . .

_o    _
o-

.s.    .  .   .   .   .   .   .   .   .   .   .
E          :    --    -   :01

.     .   .   .   .   .   .   .   .   . . .

. . . . . . . . . . .

o ...........
D

...........

o

as      0S   a   O_c
73 _

t-3 I- t- e0__t-

0

o

,a C C

Q '4

to

4
-3

0

0
0

p .  .   .   .   .   .   .   .   .   .

eq -00 o- eq -0 1 .-i

C10 "   0   -4 10  - e 0  C   0 0

lo = m " la m cq  m = 0
CO  C   -  0  "   be 00

eq -4   -4       - -

100     O O CO O CO

?  .  . .  .  . .  o

.. * _  *   cf * _

. -  . - 4

* ~ e q  0   .  0   e q  *

"4 Co10

- 0   e q  0 0 1   C O X f
- - 0 - eq

G o  rZ - (O

. . . . .
CO eq . e 0

* . . ..

0 - * t- 10

eq

t- '*  .-  .   .

CO  10'  CO*  4   0

eq o eq -

* . . . .

- - -   - =

= 4 (M - co

4 c  -4 -

J-.4  -4 . 4.4   -4  -...

o .   .  . . . . . . .   .   .   .  .  .  .  .  .  .

b  :  * . . . . . . ..   .   .,

5to                      X  e       _

t-
c-

CO eaq     C

4   e  -    eq

0 m COO

-      C

.    .   . -
--   :  1-   ..

0

o

CO

0

0

CO 10OCm eq

- ~ C

CO

-4 - 4 -   -

l            0

eq

.   I

0:   0

._

0

o~~~~~~~~~~~~~~~~~~~

0
0

0

cc

0

o

0

co
0

9-4
04

5

- me

-o

F 0
O

* e
_

me,

I

.t e
H C

5

12
5

i

4
.4

D
D

4

4

q

t
I

c
c
c
p

M. ATKI    et al.

io

s-   C O t - 0 0 t - 0>

"C.,

.= _  v  . .  . .  . ..

ts_  :4,_ _ 4_.Fm

-W o ::   -t  = 'o   =
C_     *'0030303

C 4

I C

-'0-

c 4 ; -  'do QC _   O

14.    C14      - .   * .

rCID Nt V IC &a ce
IC   0  =  = t- cq 1

Co

0

Cq

0

0
03

.4 X      Ca    . . . .

Pm  -z fl  0 >. "- ~- >     >

.-        4     " 04

EC 0

cq cq -

-4 -4 P

c C 00
C03

o _- IC 00
o ,-

-4-400

00 0003q

03

0~

00

P-

0-4 - ~- 4
l,-i I,,

i,0--   "., i ,--iI'

I?~ *.T

~ 00

: m :.ic! : :

- O-
lq  r-  t Go  ce. I-d

*0et' *010IC
.0   All

s c oQ

( -

03 P-
*00      t

0

CO-4PiCO  '0 4

t-coo    ' .t-
= it00 -  0 04

C      -

C)

0

T  *.  . "' S

0  *   ' - '

I- . B
~.~~~~'5

t  zo  - =  S co:   r
0> 0E 00

6

0

0
0

P-
P-

r
t'.

t-

cle
CO

00

0L
'0

09

40

to

00
'0

"-f=  o 'o C

Oc>s ter  tc
- 00 -

- 0'0a   0

r-
* * *0 -
0

o ~

0
- .z     r
*   .   .   .      *   *               *   -4

0

0

10-:1 h-1 0-       4 '- ~   ~

o

.o

a       I

0 z

Z3
r-

-4

U
to

0

s"  cc

0

'}  ..S

0 0.0

.0

0

eq o; o

G C2

_

tD 'S,8 S

t GO o

U

0
o.,.

p4
._=

C
04
0
0

0
CO
10

mi

CANCER OF THE S     AND

7

0 CD bQ

Zs  , o s _ _ o 5 o X r c co  q o e
e  Z .   .   .   .   .   .   .   .   .   .   .   .   .   .

| O  _  b  cee:  10_ s0 0 0 Cle

or            t

0 .s :9 1o o 0  Co IC o- rN  qD o   m 0oo

o   .   .   .   .   .   .   .   .   .   .   .   .   .   .   . . .

Nt to 0to l _o 00  ^ 0- eC a

?  =2X 0 --  eD CZ  X  _o  s s  c c

.   .   .   .   .   .   .   .   .   .   .   .   .   . .

s  e .   .   .   .   .   .   .   .   .   .   .   .   .   .   .  _
o      .e  Cl Cq 0 ko b  _D_

E4       cq cs  _                **

.   .   .   .   .   .   .   .   .   .   .   .   .  .  .

s    4 Co        -4      P- _q cz   P ++**  4

o      oe _            Ceqt _>  X

.       .   .   .   .   .   .   .   .   .   .   .   .   .*

ew~~~~~a       cn    m
*cW

a  v   ..................~~~~~~~C4 -I

O %

-0 CI

-      ,   S ~.::

?~~~

.      t X~~~~~~2 C)

S0        G)I:
0     --0 '

O)  Mo-

m           C

04 la- ?a %O t  C

. ,= 0  t

M     QqI  CD  CD

c  _X'   (

O .   G

_  e    o q: esx~~~~C

.   .   . .   . .   . . .   .   .   . .   . ....   .   -~ -  ..

..... ..........?...... ,            ,.,, , , ,  X,-,
. . . . . . .   .  .  -. .   ..   .  . .   .   .

......c                            o  . '

~ .~   .  .6 ~-0

Si

0
Zs
d

I

I
G
li

0           0~~~~~
~~~~~~~~0~1

M. ATKIN et al.

In the agricultural group, the general high level of percentages is clear. Six
out of 17 occupations, with a population of nearly 700,000, have percentages
from 131 to 249; this last figure (gardeners) is the highest of all those recorded
in this paper. The contrast between gardeners (249) and gardeners' labourers
(32) is obvious. This difference, which appears also in regard to many other
diseases, is discussed by the Registrar-General (1938, p. 79, and Table 4), p.
307), who says, "The separation of agricultural labourers including shepherds
from gardeners' and estate labourers, pea and fruit pickers was attempted in
No. 93 and 94, Tables 4D), 6A, but the very low mortality figures for Group 94
(gardeners' labourers, estate labourers, pea and fruit pickers) for every cause
suggests that the definition of these occupations on death certificates was not
as precise as on census schedules, and no reliable conclusions can be drawn from
this subdivision." One may suppose that the informant at death registration
would often call the deceased a "gardener," when the man himself would have
described himself as "gardeners' labourer" at the census. In view        of this
factor, the result of pooling the figures for the two occupations is shown in
Table II; the percentage for cancer of the skin is then 198 in place of 249.

The figures for the registered deaths as percentages of the calculated deaths
for each of the three classes (professional 47-5, mining 94-4, agricultural 142-4)
have been included in Table II; the result, of course, depends largely upon the
population of the larger groups, e.g., the figure for mining (94-4) is influenced by
the large number (466,194) of hewers and getters (percentage 97- 7).

A study of the social distribution of cancer of the skin in a large number of
occupations in England and Wales in 1930-32 has been made by Ryle and Russell
(1947).

The mortality from cancer of the skin and of the lip upon these groups of
occupations may be examined in another way (Table Il), namely by calculating
the populations which produce one case of cancer in the period in question.
(The factors used in calculating the standardized figures for deaths from cancer
of the lip shown in Table mI were derived from the census population for males
over 14 in 1931, and the published figures for deaths from cancer of the lip in
the period 1923-1943. The fact that these factors are applied to the occupational
deaths in the period 1911 to 1944 is not thought to introduce an appreciable
error.) The age distribution of the various groups must be considered, as an
occupation employing a large proportion of older people will show a high incidence
of cancer apart from    any occupational factors. The results given by the
standardized and un-standardized figures are shown in Table III. The number
of professional workers producing one death from cancer of the skin (1163; 1549)

TABLE III.-Occupational Mortality. Cancer of Skin and Lip.

Population.

Poation.    Deaths,   Population producing one death from cancer of

occupied  1911 to 1944.

ccuupied  1911 to 1944.       skin and cancer of lip.
Occupational group.  and retired,  Cancer of

aged 14        --      nstandardized.     Standardized.

and over.  Skin.  Lip.                               ip
1931 census.            Skin.   ip.     Skin.         ip.

Agiculture    .   . 1,200,397 . 2,990 1,220 . 401  878 . 516 = 1-0   1,590 =  1- 0
mirng      .    .   1,018,049 . 1,049  290 . 970  3,510 . 780 = 1-5  2,872 -= 1-8
Professional  .   .   394,302 .  339   27 . 1,163  14,000 . 1,549 = 3-0 20,644 -= 12-9
Total cf above groups. 2,612,748 . 4,378 1,537 . 597  1,700 . 660    2,167
General population  . 14,050,209 .19,087 8,481 . 736  1,657 . 736    1,657

8

CANCER OF THIE SKIN ANID LIP

is about three times as great as the corresponding figure for agriculture (401; 516)
in both the standardized and unstandardized series, while the miners take an
intermediate position (970; 780).

(b) Occupational distriWon according to anatomicd site.

The sites of cancer of the skin named on the death certificates were classified
as follows:

Lower limb
Upper limb
Trunk

Head and neck
Face
Ear

Site not stated.

The subdivision of the data into smaller groups, e.g. fingers, hand, forearm,
and so on, might seem to give more information, but the sampling errors would
be increased, and in such material the assignment to the smaller sites has not
been carried out on any uniform system; e.g. there would be no certainty that
some cancers of the fingers had not been classed as of the hand.

The numbers of cancers at these sites in the three groups of occupations, and
their percentage distribution, are given in Table IV and Fig. 2. The inferences
which can be drawn from such percentages are, of course, very limited, in view
of the effect of each item upon the others. The greatest difference between the

TABLE IV.-Occupational Distribution of Cancers of the Skin, according to Site.

Agrkulm  l occupations.      Minng occupat         Profeonal occupatioms.

Deaths    %.                aths                    Deaths. %. De
,owerlimnbs .    .    119     4 0          .     68     6 5          .   32      9 4
Tpper limbs .    .   398     13-3          .     50     4-8          .    15     4-4
runk .     .     .    105     3-5          .     65     6-2          .    33     9- 7
[ead and neck    .    167     5-6          .     66     63           .    15     4-4

ace   .    .    .   1524     51 0  73-5   .    578     55 0  .781   .   169     50-0 .69-
ar         .         505     16-9 J        .    176    16-8 J        .    52    15-3 J
ot sited    .    .    172     5- 7         .     46     4-4          .    23     6-8

2990   100-0

1049    100-0

7

.   339   100-0

upper and lower limbs is in the agricultural group, where the former gives the
higher figure, which result one might attribute to exposure to sunlight. The
percentages of head, neck and face together do not differ very much in the three
groups, (73-5, 78-1, 69-7). The face may undergo considerable exposure to
sunlight in the professional classes.

A study of the distribution of skin cancer according to site in Northern Ireland,
in which a different classification of sites is adopted, has been published by Ryle
and Russell (1947).

2. Cancer of Lip.

Fig. 1 provides a comparison of the percentages for cancer of the skin, and
of the lip, selected and arranged in the same way, except that all cancers of the
lip are included and not only those shown by occupation yielding more than 5
deaths. The number of fatal cases of cancer of the lip in the professional classes
is so small (27 in 34 years in about 400,000 men) that these have been pooled

L
U
T
B
F
E
N

Total

9

10                        M. ATKN et a.

without calculation of percentages for the individual occupations; the percentage
for the whole group is then 8-4, which is the lowest figure obtained in all the
investigations by this method carried out by some of the authors of this paper
on cancer of the skin, lung, larynx, bladder and prostate. Fig. 1 and Table II

I/"'

L.U

90

80

70

60

50

40

30

2

1

'0
0

Nor
SITED

EAR
FACE
FAC E

HEAD AND

NECK
-TRUNK

UPPER
- LIMBS

LOWER
LIMBS

NOT
SITED

EAR

FACE

HEAD AND

NECK

TRUNK

UPPER
LIMBS
LOWER
LIMBS

NOT
SITED

EAR

FACE

HEAD AND

NECK
TRUNK

UPPER
LIMBS

LOWER
LIMBS

AGRICULTURE MINING PROFESSIONAL

FIG. 2.-Occupational distrnbution of cancers of the skin according to site.

show that no one of the seven groups of coal and shale miners gives a percentage
above 100; the figures are low (38-3 to 56-0 per cent) except in one large group
which is the most exposed to light (other workers above ground, population
106,549), in which the incidence (97-5 per cent) is approximately the same as in
the general population. The numbers in the other branches of mining (metal
miners, other mines and quarries) are small for statistical purposes, but one
notes that stone miners and quarriers, many of whom must be exposed to sun-

-

-

-      -      - i

I

I

CANCER OF THE SKIN AN  LIP

light, are the only miners showing a percentage (153-4) which is above 100, and
that this figure is of the order of that found in open-air workers. Eight of the 13
agricultural occupations represented in Fig. 1 show percentages above 100, the
highest figure being 205 (shepherds).

The ratios in the last two columns of Table III show the much greater difference
between the professional classes, and the miners and open-air workers, which is
revealed by the figures for cancer of the lip in comparison with those for cancer
of the skin.

The high incidence of cancer of the lip upon civil engineers and surveyors,
in comparison with other professional workers, is shown by the following rough
calculation, though the numbers are of course very small (Table V):

TABLE V.--Cancer of the Lip in Civil Engineers and Surveyors.

Professional Occupations. Social Class I.

Deaths frm   Popu~lou producng
camir of lip.   one deat.
Civil engineering and surveying  .  24,352  .   5       .      4,870
Other profens  .     .   .     191,179   .      8       .     23,874

215,531   .      13

DISCUSSION.

Cancer of the skin.

The material summarized in Tables II and III, and in Fig. 1 shows that the
incidence of fatal cancer of the skin is high upon agricultural workers, and very
low upon the professional classes, while the miners take an intermediate position
not far removed from that of the general population. The especial liability
of the out-door workers is generally supposed to be due to exposure to sunlight
(Blum, 1948).

The results given in Table IV and Fig. 2 show that the cancers of the whole
region of neck, head, and face together, selected on the basis described above,
make up from 70 to 78 per cent of cancers of the skin in all three occupational
groups. The face is the only part of the body which is never clothed, and those
parts which are shaved receive more thorough cleansing than do any others;
these characters are common to all social classes. The most notable result of
this tabulation is the lack of any great difference between the three groups.

Another factor which may be of considerable importance is that the cosmetic
effect of cancer of exposed portions of the skin and of the lip may attract attention
sooner in richer subjects. Ingram (1947), in a comment on the paper by Ryle
and Russell (1947), says: "Prognosis in skin cancer depends largely upon the
size of the lesion, and negligence is probably an important factor in relation to
death from this cause. I should, in the ordinary course of events, expect neglect
of a symptomless lesion to be higher in unskilled workers and labourers (IV and
V of the Registrar-General's social classes) than in the other groups." Obviously
one cannot assess the quantitative importance of this difference.

Data in equal detail for the distribution of skin cancer in married women
whose husbands follow these occupations would be of great interest. The
Decennial Supplement for 1931 shows that the comparative mortality from

11

M. ATKIN et al.

cancer of the skin upon the Registrar-General's five social classes is similar i

men and married women (Table VI), and the same phenomenon is shown by

TABLE VI.-Social Distrintion of Cancer of the Skin Among Men

and Married Women.

Cancer of Skin, 1930-1932. Standardized Mortality Ratio.

Deennil Suppkment, 1931.                 Ryle and RuselL

?   A                                ~~~~~~~~~~~~~~~~~~~~~~~~~~A

Age 35 to 65.                    Age 20 and upward.
Soal das                             Group.

I and II  .    72    .     92    .    A     .     70    .     75

III    .     95    .     89    .    B     .    102    .    116
IV     .    116    .  . 114    .     C    .    160    .    152
V      .    132   .    131    .

3 groups of occupations, A, B and C, showing a similar descent in the social scale,
which were studied by Ryle and Russell (1947), using the Registrar-General's
figures.

Such material provides a most valuable indication of environmental, as
distinct from occupational, factors. For a discussion of this matter see p. 3 of
the Decennial Supplement, 1931, Part IIa. The Registrar-General (p. 46)
concludes that in the case of cancer of the skin,"    . . . most of the
differentiation arises from selective factors or from environmental conditions in
the homes rather than from the occupations themselves  . . . although the
employment of a proportion, largest no doubt in Classes V and V, of married
women in textile occupations may be partly responsible for the mortality
gradient for skin cancer."
Cancer of the lip.

Material obtained from death certificates does not allow one to distinguish
between cancers arising from the upper or lower lip, or from different portions of
either of these. For a discussion of aetiological factors see Lane-Claypon (1930),
and Willis (1948), who says (p. 294) ".   . . The great preponderance of
tumours of the lower lip is explained as due to direct exposure of its muco-
cutaneous junction to sunlight, while the corresponding part of the upper lip
is relatively shaded." Some factor in addition to exposure to sunlight is required
to explain the differences shown in Table VII. Smoking, and especially pipe
smoking, is, of course, regarded as another possible factor in the production of
cancer of the lip. During the earlier part of the period (1911 to 1944) cigarette
smoking was practically restricted to the richer classes, which produce fewest
cancers of the lip; data on the change in this matter in recent years have been
given elsewhere (Kennaway and Kennaway, 1947). The data collected by
Lane-Claypon (1930) from the literature, summarized in Table VII, show the
great preponderance of cancers of the lower lip, especially in males. Of 4839
cases where the sex was stated, 93-5 per cent were male. Of 3763 cases where the
site was stated, the lower lip was involved in 94-2 per cent. Table VII records
the data from 2676 cases in which sex and site for each individual were stated.

Lane-Claypon's data are derived from published series, which might be
liable to some kind of selection, but the greater incidence upon males is con-

12

CANCER OF THE SKIN AND LIP

TA.B    VI.-Cancer of the Lip (Lane-Claypon, 1930).

Malea                  Females

Number.    .       Nmber.  %.   % of Tota.
Lower lip    .   .    .   .    .    .   2378   96-4    .   160    76-2    6-3
Upperlip     .   .    .   .    .    .     88    3-6    .    50    23-8   36- 2

l..Totals  {Fml              .   2466     100   .    210    100    7- 8

Female    .   .    .   210

2676

firmed by the unselected figures of the Registrar-General.  In the years 1923-43
inclusive there were in England and Wales 5897 deaths attributed to cancer of
the lip, of which 506, or 8-6 per cent, were in women.

The high incidence of cancer of the lip upon outdoor workers is shown by
figures for England and Wales for 1911 to 1913, obtained by Lane-Claypon
(1930) from Dr. Stevenson of the General Register Office (Table VIII).

TABLE VIII.-Cancer of Lip. Mals. England and Wales, 1911-13. (Lane-

Cilaypon, 1930).

Death rate
Deaths.      per million

per annul.

All occupied and retired males   .     .    750      .    21- 2
Agricultural labourers      .    .     .    128      .    79- 8
Farmers    .    .     .     .    .     .     56     .     55-0

Comparison of cancer of the skin and of the lip.

The data given in Fig. 1 and Table II, and especially in the two last columns
of Table III, show differences between the three occupational groups which are
greater in the case of cancer of the lip than in that of cancer of the skin. These
results, derived from certain selected occupations, may be compared with those
given by the Registrar-General for the social distribution of these cancers in the
whole male population (Table IX), which show a much steeper social gradient
in the case of cancer of the lip than in that of cancer of the skin.  The results

TABLrE IX.--Comparative Social Incidence of Cancer of the Skin and of the Lip.

(Regi8trar-General, 1927, 1938.)
Standardized Mortality Ratio.*

Males. England and Wales.

Social class  .  .  .   I.          II.        III.         IV.          V.

Ages 20-65. 1921-23.

Cancer of skin .  .    63     .     73    .     100    .    120          150
Cancer of lip  .  .    30     .     50    .      70    .    140     .    170

Ages 35-65. 1930-32.

Cancer of skin .  .   t(59)   .     75    .     95     .    116     .    133
Cancer of lip  .  .   t-      .     56          68     .    147     .    183

* Figures for 1921-23 are based on those for all occupied and retired males, while those for
1930-32 are based on those for all males (i.e. including unoccupied).

t Ratios based on less than 20 registered deaths are shown in brackets, or if both the registered
and standard deaths are less than ten, omitted.

13

M. ATKN et al.

reported in the present paper are an attempt to carry this analysis further by a
study of occupations providing different environmental conditions. The ratios
of the populations producing one death from cancer of the skin, or of the lip
(Table III), namely,

Agriculture.  Mining.fsonal

occupations.

Cancer of skin                  1 .  1- 0  .  1- 5  .    3- 0
Canceroflip      .    .    .    1-0         1- 8   .    12 9

indicate that in cancer of the lip, some anti-carcinogenic factors, very much more
powerful than the protection from sunlight which the miner's life affords, are
active among the professional classes. Ingram's "negligence factor" (see
above) must be borne in mind, and also the susceptibility of the lip to defective
supply of vitamin B2 (riboflavin).

The predominantly high incidence of cancer of the skin upon agricultural
workers is shown by the fact that these occupations employ 1,200,397 men,
and of these, 1,021,857,-or 91 per cent, follow occupations in which the percentage
for skin cancer is above 100, while the corresponding population in the case of
cancer of the lip is 489,526, or 43 per cent. This difference is due to the low
incidence of cancer of the lip on the large populations of gardeners and farmers
(total 545,717), and it is noteworthy that these occupations show the greatest
difference in the incidence of these two forms of cancer. Of the 14 agricultural
occupations which produce any cancers of the lip, 11 show agreement in regard
to cancer of the skin and of the lip in that both percentages are either below,
or above, 100. This suggests that the aetiological factors are to some extent
the same in some occupations. The 3 occupations showing discrepancy in this
respect are

Registered per cent of calculated deaths.
Cancer of sin.    Cancer of Lip.

Gardeners and gardeners' labourers    198-0      .      66- 6
Farmers   .     .    .    .    .      113-6      .      71- 7
Foresters and woodmen     .    .       98 4      .     128 6

Registered deaths as percentages of calculated deaths for skin cancer in the
three main occupational groups considered (professional, mining, agricultural)
are fairly evenly spread about the figure of 100 (47-5, 94-4, 142-4), but the
corresponding percentages for cancer of the lip are much lower (though in the
same order) (8-4, 59-8, 1094). The fact that the highest of these is 109-4 raises
the question, "What are the other occupational groups which show a percentage
of over 100 for cancer of the lip?"  The investigation will be pursued in this
direction.

SUMMARY.

A study has been made of the death certificates of cases of cancer of the skin,
and lip, in males engaged in agriculture, mining and professional occupations
in England and Wales during 1911 to 1944. The mortality is highest in
agricultural workers, and lowest in the professional classes, while the miners
take an intermediate position. The special liability of outdoor workers to
cancer of the skin and lip is generally attributed to exposure to sunlight, but the

14

CANCER OF THE SKIN AND LIP                         15

data given here show that other factors are involved also. The incidence upon
the coal miner is of especial interest in view of the cleanliness secured by his
daily bath, and of his minimal exposure to sunlight. In occupations in which
88 per cent of the coal-mining population are engaged the liability to cancer
of the skin is less than it is in the general population. Only one occupation of
Social Class I (civil engineers and surveyors) shows a mortality from cancer of
the skin higher than that in the whole population; hence these results illustrate
the social distribution of cancer of exposed sites discovered by Stevenson. The
comparative distribution of cancer upon the various parts of the skin does not
show any great differences in the three groups; from 70 to 80 per cent of these
cancers are situated on the head and neck. The difference between the pro-
fessional classes on the one hand, and the miners and agricultural workers on the
other, is much greater in the case of cancer of the lip than in that of cancer of
the skin. The agricultural occupations in which the ratio of registered to
calculated deaths is greater than 1 contain 91 per cent of the population in the
case of cancer of the skin, and 43 per cent in that of cancer of the lip. Only
27 deaths from cancer of the lip were recorded in 34 years in men of the profes-
sional classes, who number about 400,000. Various aetiological factors are
discussed.

We are indebted to the Registrar-General, and to the Factory Department of
the Ministry of Labour and National Service, for the data considered in this
paper, and to members of the staff of the General Register Office for advice.
We wish to express our gratitude to the British Empire Cancer Campaign, the
Anna Fuller Fund and the Jane Coffin Childs Memorial Fund for Medical
Research, forgenerous grants. We wish to thank Dr. W. T. Russell for criticism.

REFERENCES.

AGN-EW, D.-(1947) ' Bevin Boy,' London (Allen & Unwin).
BLUM, H. F.--(1948) J. nat. Cancer Inst, 9, 247.

CON-AD, K. K., A_N-D BRASDFORD-HILT. A.--(1939) Amer. J. Cancer, 36, 83.
LNGRAM, J. T.--(1947) Brit. med. J., ii, 33.

KENNN-AWAY. E. L., AND KE_ NxWAY. N. M.--(1947) Brit. J. Cancer, 1,260.

KEN-AWAY, N. M., AND KENN-AWAY, E. L.--(1936) J. Hyg., Camb., 36, 236.
KN.owLEs, R. B.--(1944) Brit. med. J., ii, 430.

LAN_E-CLA-PO_s-, J. E.--(1930) 'Report on Cancer of the Lip, Tongue and Skin.'

Report No. 59. Ministrv of Health. London.

Registrar-General's Decennial Supplement. England and Wales, 1921. H.M1. Sta-

tionerv Office. 1927.

Registrar-General's Decennial Supplement. England and Wales, 1931. Part HIa.

Occupational Mortality. H.M. Stationery Office. 1938.

Registrar-General's Statistical Review of England and Wales. Tables and Text.

London. H.M. Stationery Office.

R-iLE, J. A., AND RUSSELL, W. T.-(1947) Brit. med. J., i, 873.
SEAW, S.--(1946) ' Guttersnipe,' London (Sampson Low).
STEvEN_-sON, T. H. C.-(1923) Biometrika, 15, 382.

Wn.Tus, R. A.-(1948) 'Pathology of Tumours'. London (Butterworth & Co.).